# Factores asociados con los microorganismos causantes de infección de las vías respiratorias inferiores en niños de un hospital privado de Lima (Perú), 2019-2022

**DOI:** 10.7705/biomedica.7502

**Published:** 2026-04-28

**Authors:** Alexander J. de Mello Dutra, Gabriele Oliveira, Anderson N. Soriano-Moreno, Jorge Quinto, Fernando Bobadilla, Nelson Diaz, Jorge Alave

**Affiliations:** 1 Escuela Profesional de Medicina Humana, Universidad Peruana Unión, Lima, Perú Universidad Peruana Unión Escuela Profesional de Medicina Humana Universidad Peruana Unión Lima Perú; 2 Unidad de Investigación Clínica y Epidemiológica, Escuela de Medicina, Universidad Peruana Unión, Lima, Perú Universidad Peruana Unión Unidad de Investigación Clínica y Epidemiológica, Escuela de Medicina Universidad Peruana Unión Lima Perú; 3 Departamento de Pediatría, Clínica Good Hope, Lima, Perú Clínica Good Hope Departamento de Pediatría Clínica Good Hope Lima Perú; 4 Departamento de Medicina Interna, Clínica Good Hope, Lima, Perú Clínica Good Hope Departamento de Medicina Interna Clínica Good Hope Lima Perú; 5 Departamento de Medicina, Facultad de Medicina, Universidad Peruana Unión, Lima, Perú Universidad Peruana Unión Departamento de Medicina Facultad de Medicina Universidad Peruana Unión Lima Perú

**Keywords:** infecciones del sistema respiratorio, infecciones por coronavirus, coinfección, niño, Perú., Respiratory tract infections, coronavirus Infections, coinfections, child, Perú.

## Abstract

**Introducción.:**

La infección de las vías respiratorias inferiores es la causa principal de muerte infantil en el mundo. Los agentes etiológicos mostraron variación estacional, geográfica y relacionada con la pandemia de la COVID-19.

**Objetivo.:**

Determinar los factores asociados con los agentes patógenos y sus manifestaciones clínicas en niños con infección de las vías respiratorias inferiores, hospitalizados antes, durante y después de la pandemia en un hospital privado de Lima, Perú.

**Materiales y métodos.:**

Se llevó a cabo un estudio retrospectivo de cohorte de pacientes menores de 13 años hospitalizados por infecciones de las vías respiratorias inferiores. Se utilizaron dos pruebas de la reacción en cadena de la polimerasa (PCR) múltiple, CLART Fast PneumoVir™ y PneumoCLART Bacteria™ para identificar los agentes patógenos. Se determinaron los factores asociados con la hospitalización prolongada según los agentes patógenos que resultaron positivos y las manifestaciones clínicas generales.

**Resultados.:**

Se analizaron 612 registros de pacientes. La edad promedio fue de 2,89 años. Se identificó el virus sincitial respiratorio en el 33,66 % (206/612) de los casos, el de la influenza A en el 26,31 % (161/612) y *Haemophilus* spp. en el 19,28 % (118/612). Antes de la pandemia de COVID-19, el 48,87 % (217/444) de los pacientes tuvo un agente etiológico viral y, en la después de la pandemia, el 87,78 % (115/131). En la prepandemia, el 17,34 % (77/444) tuvo más de un microorganismo, siendo frecuentes las coinfecciones del virus sincitial respiratorio con *Haemophilus* spp. (10,78 %) (66/612) y del virus sincitial respiratorio con el virus de la influenza A (8,82 %) (54/612). Además, la identificación del virus sincitial respiratorio se asoció con una estancia hospitalaria de 6 o más días (p = 0,005).

**Conclusiones.:**

En este estudio, se encontró que los agentes etiológicos virales fueron predominantes antes y después de la pandemia. Las coinfecciones entre bacterias y virus fueron frecuentes. El virus sincitial respiratorio fue el más frecuente y se asoció con coinfecciones y estancia hospitalaria prolongada.

## Abstract (en)

**Introduction.:**

Lower respiratory tract infection is the leading cause of childhood death wordwide. Etiological agents showed seasonal, geographic, and pandemic-related variation.

**Objective.:**

To determine the clinical manifestations of respiratory pathogens in children hospitalized due to lower respiratory tract infection before, during, and after the pandemic in a private hospital in Lima, Perú.

**Materials and methods.:**

This is a retrospective cohort study of patients under 13 years of age hospitalized with lower respiratory tract infection. Two multiplex polymerase chain reaction (PCR) tests, CLART Fast PneumoVir™ and PneumoCLART Bacteria®, were used to identify pathogens. We identified factors associated with prolonged hospitalization based on general characteristics and the presence of pathogens.

**Results.:**

A total of 612 patient records were analyzed. The average age was 2.89 years. Respiratory syncytial virus was identified in 33.66% (206/612) of cases, influenza A in 26.31% (161/612), and *Haemophilus* sp. in 19.28% (118/612). Before the COVID-19 pandemic, 48.9% of patients had a viral etiologic agent, and 87.78% (115/131) had one after the pandemic. During the pre-pandemic period, 17.34% (77/444) of patients had more than one microorganism, with frequent coinfections of respiratory syncytial virus with *Haemophilus* sp. (10.78%) (66/612) and respiratory syncytial virus with influenza A virus (8.82%) (54/612). Additionally, the identification of respiratory syncytial virus was associated with prolonged hospital stays of more than 6 days (p = 0.005).

**Conclusions.:**

This study found that viral etiologic agents were predominant before and after the pandemic. Furthermore, coinfections between bacteria and viruses were frequent. The respiratory syncytial virus was the most common and was associated with coinfections and prolonged hospital stays.

La infección de las vías respiratorias inferiores las afecta desde la tráquea hasta los alvéolos. En la población pediátrica, la tos es el principal síntoma, asociándose con disnea, expectoración, dolor torácico, rinorrea, estornudo y sibilancias ^1^.

Generalmente, los virus son los agentes patógenos que ocasionan con mayor frecuencia la infección respiratoria aguda. Aproximadamente, el 70 % de todas las infecciones respiratorias agudas de tipo viral son ocasionadas por virus respiratorio sincitial, rinovirus-enterovirus, metapneumovirus humano, virus de la parainfluenza, virus de influenza, coronavirus humano, adenovirus o bocavirus humano ^2^.

Entre los agentes bacterianos, los más frecuentes son *Streptococcus pneumoniae, Staphylococcus aureus, Klebsiella pneumoniae y Haemophilus influenzae B*^1^.

Otra causa importante de infecciones respiratorias pediátricas bacterianas lo constituyen los agentes patógenos atípicos, como *Mycoplasma pneumoniae, Chlamydophila pneumoniae* y *Legionella pneumophila*^3^.

Asimismo, es común que pacientes con infecciones respiratorias agudas virales tengan coinfecciones virales o bacterianas ^4^^,^^5^.

En Latinoamérica, pocos estudios se han realizado con el objetivo de determinar las características de las infecciones respiratorias durante la pandemia y la pospandemia por la COVID-19 (*coronavirus disease*-2019). En un estudio en México, se observó una disminución de la incidencia de las infecciones respiratorias agudas en la primera mitad del 2021. En Brasil, se observó una disminución de los cuadros de bronquiolitis aguda en el 2020, lo cual, según el estudio, se debió a las medidas de distanciamiento social ^5^^,^^6^.

Según el boletín de la Dirección General de Epidemiología del Perú de las infecciones respiratorias agudas en pacientes menores de cinco años, en el 2019 el número de casos de este tipo de infección fue de 2 501 079. Además, se presentaron 25 617 casos de neumonía; 9274 hospitalizaciones y 192 defunciones, con un índice de letalidad del 0,75 % durante el 2020.

Estos números disminuyeron en el 2022 a 743 558 casos de infecciones respiratorias agudas, 7466 casos de neumonía, 2401 hospitalizaciones y 96 defunciones, con un índice de letalidad del 1,29 %.

Según los últimos reportes, en el 2023, estos valores volvieron a elevarse con 2 164 520 casos de infecciones respiratorias agudas, 31 771 casos de neumonía, 9932 hospitalizados y 248 defunciones, con un índice de letalidad del 0,78 %. Según el estudio, una de las posibles causas de este aumento respecto al año anterior podría ser el cambio climático que se presentó en varias zonas del territorio peruano, y afectó a los niños menores de 5 años ^7^.

Durante la pandemia por la COVID-19, las múltiples estrategias de prevención implementadas, como el uso obligatorio de mascarillas, la promoción de la higiene de las manos y el distanciamiento social en los espacios públicos, ayudaron a evitar la infección por otros agentes patógenos respiratorios ^8^.

Por ejemplo, en un estudio realizado en el norte de España en población pediátrica en 2018-2019 y 2020-202, se observó una ausencia casi total de virus respiratorios estacionales comunes, como el virus sincitial respiratorio, el rinovirus, el enterovirus, el metaneumovirus, la parainfluenza, la influenza, el coronavirus y el adenovirus ^9^.

En Rusia, se observó una variabilidad drástica de las infecciones respiratorias agudas entre niños en tres períodos:

1. en el de 2019-2020, los principales agentes circulantes fueron el virus de la influenza, el virus sincitial respiratorio y el de la parainfluenza;
2. en el de 2020-2021, predominaron el metaneumovirus, el rinovirus y el coronavirus, con una incidencia mucho menor que la de la COVID-19; además, hubo una acentuada disminución del virus sincitial respiratorio y ausencia del virus de la influenza;
3. durante el último periodo de investigación (2021-2022), los más numerosos fueron el virus sincitial respiratorio y la COVID-19; al mismo tiempo, hubo un aumento de la incidencia de rinovirus y un resurgimiento de la influenza ^10^.

En México, los indicadores nacionales demostraron una reducción de la incidencia de la infección por el virus de la influenza tras la implementación de las estrategias para disminuir la transmisión de la COVID-19 ^11^.

En Colombia, se reportó baja incidencia de infección por rinovirus, enterovirus, virus sincitial respiratorio y adenovirus, durante la segunda, la tercera y la cuarta olas de la pandemia ^12^.

Teniendo en cuenta todo lo anterior, este estudio tuvo como objetivo determinar los factores asociados con los microorganismos causantes de infecciones de las vías respiratorias inferiores, identificados mediante pruebas moleculares, y las manifestaciones clínicas de los pacientes pediátricos hospitalizados del 2019 al 2022, antes, durante y después de la pandemia por COVID-19, en un hospital privado de Lima (Perú).

## Materiales y métodos

### Diseño y población de estudio

Se llevó a cabo un estudio retrospectivo de cohorte, utilizando la información clínica y de laboratorio de los pacientes pediátricos admitidos en la unidad de hospitalización pediátrica de la Clínica *Good Hope*, entre el 1^o^ de enero del 2019 y el 31 de diciembre de 2022.

Este hospital privado está ubicado en el distrito de Miraflores, perteneciente a la provincia y departamento de Lima, capital del Perú. Se incluyeron todos los registros de los pacientes menores de 13 años admitidos con el diagnóstico clínico de infección de las vías respiratorias inferiores, que requirieron hospitalización.

### Variables

Se evaluaron las siguientes variables sociodemográficas:

1. sexo (masculino o femenino),
2. edad (años),
3. distrito de procedencia de Lima según la zona (centro, norte, sur, oeste, este),
4. estacionalidad al momento de la hospitalización (primavera, verano, otoño o invierno),
5. periodos relacionados con la pandemia, según el boletín epidemiológico del Perú: prepandemia, antes del 16 de marzo del 2020; pandemia, entre el 2020 y el 2022, y pospandemia, posterior al 4 de abril del 2022.

Las variables clínicas incluidas fueron: fiebre, rinorrea, otalgia, tiraje subcostal, tiraje intercostal, sibilancias, roncus, odinofagia, hiporexia, estornudos, tos, vómitos, disnea o convulsiones. La gravedad se determinó según los criterios de la Organización Mundial de la Salud (OMS) y si fue necesaria la hospitalización prolongada, definida como «tiempo mayor que la media del tiempo de hospitalización (5,96 días) de los pacientes».

Los pacientes con infección de las vías respiratorias inferiores se dividieron en dos grupos: 1) aquellos que cumplían con los criterios de gravedad de la OMS (infección respiratoria aguda con antecedentes de fiebre mayor de 38 grados y tos, síntomas establecidos en los últimos 10 días) y 2) los que no los cumplían, según lo descrito en la historia clínica.

### Detección de los agentes patógenos

Se practicó una prueba de reacción en cadena de la polimerasa en tiempo real (qPCR o PCR cuantitativa) de muestras de hisopados nasofaríngeos para virus y bacterias respiratorias de GENOMICA’s Clinical Array Technology, CLART™.

Para la detección de virus, se usó la prueba CLART Fast PneumoVir™, que permite la detección de: adenovirus, bocavirus, coronavirus, enterovirus, Hl NI, H3N2, de la influenza A-C, metapneumovirus A-B, de la parainfluenza 1-4, rinovirus y virus sincitial respiratorio A-B.

Para la detección de bacterias, se utilizó el panel de PneumoCLART bacteria™, que permite la detección de: Bordetella spp. (*B. parapertussis, B. pertussis, B. holmesii, B. bronchispetica*), *Haemophilus* spp. (*H. influenzae*), *Moraxella catarrhalis, M. pneumoniae, S. pneumoniae y S. aureus*.

Todas las pruebas fueron practicadas por personal capacitado del laboratorio, aplicando medidas higiénicas y de bioseguridad.

### Extracción de datos

La información de cada episodio de hospitalización por infección de las vías respiratorias inferiores se obtuvo a partir de la revisión de las historias clínicas electrónicas entre enero y diciembre del 2023 en las instalaciones de la Clínica *Good Hope*.

### Análisis estadístico

Describimos las características generales de los pacientes y los agentes patógenos de la muestra general, utilizando frecuencias relativas y absolutas para las variables categóricas y, medidas de tendencia central y dispersión, para las numéricas. Se crearon gráficos de tiempo para visualizar el número de casos por mes. También, se calcularon los porcentajes de coinfección.

Se construyeron líneas de tiempo por agente patógeno para ubicar de manera gráfica las frecuencias mensuales de positividad para virus y bacterias respiratorias. Además, se diseñó una matriz de calor para graficar las proporciones de coinfección entre pares de agentes patógenos. Se evaluaron los factores asociados con la hospitalización prolongada según las características generales considerando las variables de edad, sexo, estación del año, distrito de procedencia y periodo relacionado a pandemia y los agentes patógenos que resultaron positivos.

Se utilizaron las pruebas de X^2^, Fisher y Wilcoxon para determinar diferencias estadísticamente significativas. Se usó la regresión de Poisson con varianza sólida para calcular las razones de prevalencia-con sus intervalos de confianza al 95 % sin ajustar y ajustados-de la asociación entre las características generales de los pacientes, los signos y síntomas y la hospitalización prolongada.

La regresión ajustada incluyó todas las variables que, en la regresión sin ajustar, tuvieron p menor de 0,20. Las variables con p menor de 0,05, tanto en las tablas comparativas como en la regresión ajustada, se consideraron como estadísticamente significativas. El análisis se realizó con el programa estadístico R, versión 4.

### Aspectos éticos

El presente estudio fue aprobado por el Comité de Ética de la Universidad Peruana Unión (2022-CE-FCS - UPeU 142) y por el Comité de Docencia e Investigación de la Clínica *Good Hope*.

## Resultados

Durante el periodo de estudio, 612 pacientes (306 de sexo femenino y 306 de sexo masculino) fueron hospitalizados por infección de las vías respiratorias inferiores. La edad promedio fue de 2,89 años con una desviación estándar (DE) de 2,80 y el tiempo promedio de hospitalización fue de 5,96 días (DE: 2,97).

El otoño fue la época del año en que se observaron más casos, con 212/612 (34,6 %) hospitalizaciones y la zona más afectada fue Lima oeste, con 301/612 casos (49,2 %).

Antes de la pandemia, se hospitalizó el 72,5 % (444/612) de los pacientes, durante la pandemia, el 6 % (37/612) y, después de la pandemia, el 21,4 % (313/612) (cuadro 1).

**Cuadro 1:** Características generales de la muestra (N = 612) en relación con los días de hospitalización

Variable		Días de hospitalización [n (%)]						
		N*		≤6 (n = 426)		> 6 (n = 186)		p**
Número de días hospitalizado		5,96	(2,67)					
Edad (años)		2,89	(2,80)	2,96	(2,79)	2,74	(2,84)	0,230
Sexo								0,292
	Femenino	306/612	(50,0)	207/306	(67,6)	99/306	(32,4)	
	Masculino	306/612	(50,0)	219/306	(71,6)	87/306	(28,4)	
Estación del año								0,125
	Invierno	153/612	(25,0)	111/153	(72,5)	42/153	(27,5)	
	Otoño	212/612	(34,6)	139/212	(65,6)	73/212	(34,4)	
	Primavera	145/612	(23,7)	97/145	(66,9)	48/145	(33,1)	
	Verano	102/612	(16,7)	79/102	(77,5)	23/102	(22,5)	
Distrito								0,572
	Lima centro	89/612	(14,5)	64/89	(71,9)	25/89	(28,1)	
	Lima este	41/612	(6,7)	27/41	(65,9)	14/41	(34,1)	
	Lima norte	41/612	(6,7)	32/41	(78,0)	9/41	(22,0)	
	Lima oeste	301/612	(49,2)	211/301	(70,1)	90/301	(29,9)	
	Lima sur	140/612	(22,9)	92/140	(65,7)	48/140	(34,3)	
Periodo								<0,001
	Prepandemia	444/612	(72,5)	288/444	(64,9)	156/444	(35,1)	
	Pandemia	37/612	(6,0)	25/37	(67,6)	12/37	(32,4)	
	Pospandemia	131/612	(21,4)	113/131	(86,3)	18/131	(13,7)	

* Media (desviación estándar, DE)

** Prueba de X^2^ de independencia; test exacto de Fisher y prueba de Wilcoxon

Se logró identificar un microorganismo viral o bacteriano mediante los métodos moleculares ya mencionados en 415 (67,81 %) del total de 612 pacientes. De ellos, 248 (59,7 %) fueron virus, 49 (11,8 %), bacterias y, 118 (28,4 %), coinfecciones virus-bacteria.

Los microorganismos identificados con mayor frecuencia fueron: el virus sincitial respiratorio (33,7 %) (206/612), el de la influenza A (26,3 %) (161/612) y *H. influenzae* (19,3 %) (118/612). La frecuencia del de la influenza A y la B, en conjunto, fue de 35,1 % (215/612).

Por otro lado, el enterovirus (0,2 %) (1/612), el adenovirus (0,3 %) (2/612) y *S. pneumoniae* (0,5 %) (3/612) fueron los agentes patógenos identificados con menor frecuencia (cuadro 2).

**Cuadro 2.** Frecuencia de los agentes patógenos (N = 612)

Agente patógeno	n(%)	
Virus sincitial respiratorio	206	(33,7)
Virus de la influenza A	161	(26,3)
*Haemophilus influenzae*	118	(19,3)
Virus de la influenza B	54	(8,8)
*Mycoplasma pneumoniae*	43	(7,0)
*Moraxella catharralis*	25	(4,1)
Bocavirus	21	(3,4)
Virus de la parainfluenza	20	(3,3)
Metapneumovirus	16	(2,6)
*Chlamidia tracomatis*	11	(1,8)
Rinovirus	5	(0,8)
*Streptococcus pneumoniae*	3	(0,5)
Coronavirus	3	(0,5)
Adenovirus	2	(0,3)
Enterovirus	1	(0,2)

### Microorganismos asociados con la coinfección

De los 612 pacientes, en 118 (19,2 %) se identificó infección por, al menos, dos microorganismos.

**Figura 1.**
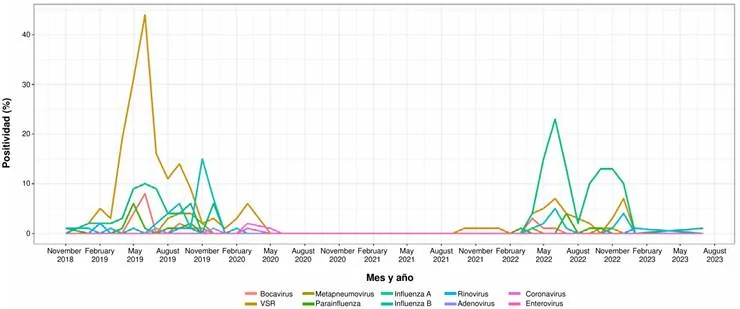
Porcentajes de positividad de virus, Clinica *Good Hope*

**Figura 2.**
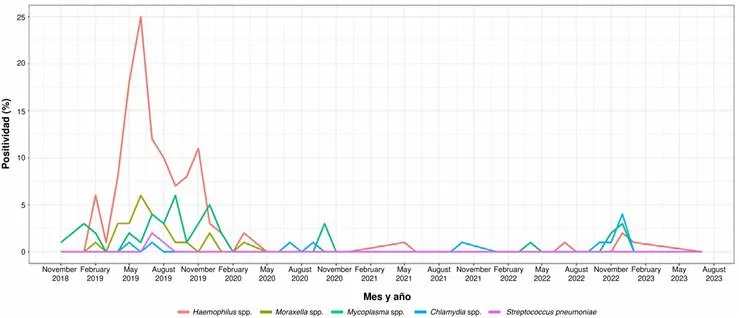
Porcentajes de positividad de bacterias, Clínica *Good Hope*

Entre los agentes patógenos, el virus sincitial respiratorio fue el que tuvo la mayor frecuencia de coinfección: 10,78 % (66/612) con *H. influenzae*; 8,82 % (54/612) con metapneumovirus; 8,82 % (54/612) con influenza A; 2,45 % (15/612) con *M. catarrhalis*; 2,12 % (13/612) con influenza B; 1,8 % (11/612) con *M. pneumoniae*; y 1,31 % (8/612) con el virus de la parainfluenza (1,31 %) (8/612) (figuras 1, 2 y 3). 

**Figura 3.**
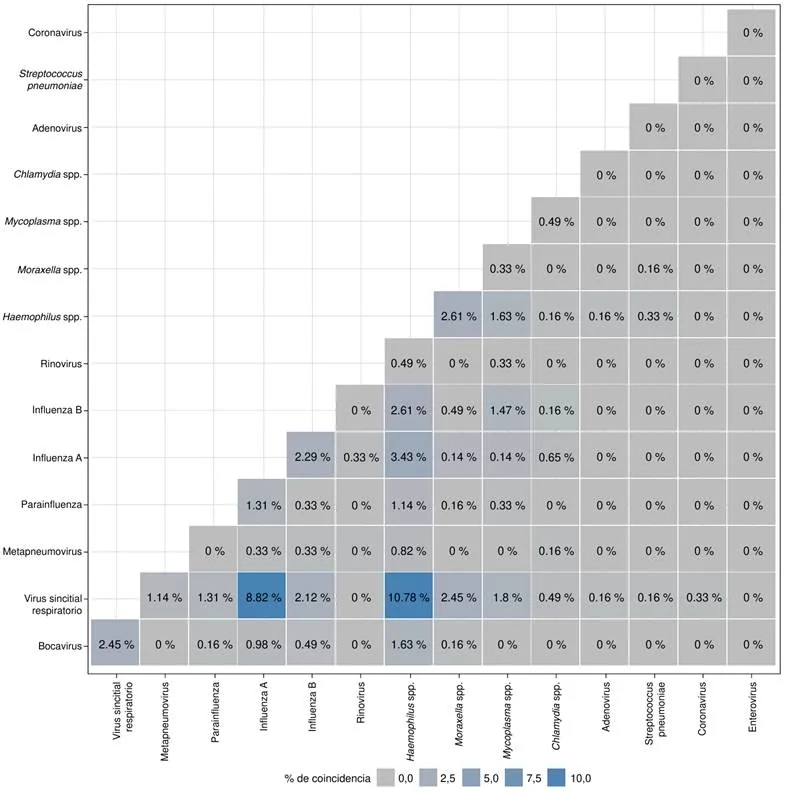
Porcentajes de coincidencia de positividad entre virus y bacterias, Clinica *Good Hope*

### Hospitalización prolongada

Entre las variables observadas, se encontró asociación entre el tiempo prolongado de hospitalización y el tiraje subcostal e intercostal (p < 0,001 y p = 0,020, respectivamente) (cuadro 3). Entre los agentes patógenos frecuentes que se asociaron con hospitalización prolongada, se destaca el virus sincitial respiratorio (p = 0,004) y *M. pneumoniae* (p < 0,001) (cuadro 4).

**Cuadro 3.** Criterios, signos y síntomas asociados con la hospitalización (N = 612)

Criterios, signos y síntomas		Días de hospitalización				
		≤ 6 (n = 426		> 6 (n = 186)		p**
		n (%)		n (%)		
Criterios OMS						
	No	139/185	(75,1)	46/185	(24,9)	0,050
	Sí	287/427	(67,2)	140/427	(32,8)	
Fiebre						
	No	192/261	(73,6)	69/261	(26,4)	0,067
	Sí	234/351	(66,7)	117/351	(33,3)	
Rinorrea						
	No	240/345	(69,6)	105/345	(30,4)	0,979
	Sí	186/267	(69,7)	81/267	(30,3)	
Disnea						
	No	180/244	(73,8)	64/244	(26,2)	0,068
	Sí	246/368	(66,8)	122/368	(33,2)	
Convulsiones						
	No	424/610	(69,5)	186/610	(30,5)	> 0,999
	Sí	2/2	(100,0)	0/2	(0,0)	
Otalgia						
	No	422/611	(69,5)	185/611	(30,5)	> 0,999
	Sí	4/5	(80,0)	1/5	(20,0)	
Diarrea						
	No	409/585	(69,9)	176/585	(30,1)	0,443
	Sí	17/27	(63,0)	10/27	(37,0)	
Vómitos						
	No	343/504	(68,1)	161/504	(31,9)	0,071
	Sí	83/108	(76,9)	25/108	(23,1)	
Tos						
	No	9/14	(64,3)	5/14	(35,7)	0,769
	Sí	417/598	(69,7)	181/598	(30,3)	
Estornudos						0,275
	No	413/590	(70,0)	177/590	(30,0)	
	Sí	13/22	(59,1)	9/22	(40,9)	
Hiporexia						0,571
	No	414/597	(69,3)	183/597	(30,7)	
	Sí	12/15	(80,0)	3/15	(20,0)	
Odinofagia						0,082
	No	407/590	(69,0)	183/590	(31,0)	
	Sí	19/22	(86,4)	3/22	(13,6)	
Roncus						0,914
	No	266/383	(69,5)	117/383	(30,5)	
	Sí	160/229	(69,9)	69/229	(30,1)	
Sibilancias						0,062
	No	202/275	(73,5)	73/275	(26,5)	
	Sí	224/337	(66,5)	113/337	(33,5)	
Tiraje subcostal						<0,001
	No	371/504	(73,6)	133/504	(26,4)	
	Sí	55/108	(50,9)	53/108	(49,1)	
Tiraje intercostal						0,020
	No	409/579	(70,6)	170/579	(29,4)	
	Sí	17/33	(51,5)	16/33	(48,5)	

* Prueba de la suma de rangos de Wilcoxon; prueba de x^2^ de independencia; test exacto de Fisher

**Cuadro 4:** Agentes patógenos asociados con la hospitalización

Agente patógeno		Días de hospitalización				
		≤ 6 (n = 426)		> 6 (n = 186)		p*
Bocavirus						0,206
	No	414/591	(70,1)	177/591	(29,9)	
	Sí	12/21	(57,1)	9/21	(42,9)	
Virus sincitial respiratorio						0,004
	No	298/406	(73,4)	108/406	(26,6)	
	Sí	128/206	(62,1)	78/206	(37,9)	
Metapneumovirus						0,787
	No	414/596	(69,5)	182/596	(30,5)	
	Sí	12/16	(75,0)	4/20	(25,0)	
Virus de la parainfluenza						0,969
	No	412/592	(69,6)	180/592	(30,4)	
	Sí	14/20	(70,0)	6/20	(30,0)	
Virus de la influenza A						0,113
	No	306/451	(67,8)	145/451	32,2)	
	Sí	120/161	(74,5)	41/161	(25,5)	
Virus de la influenza B						0,898
	No	388/558	(69,5)	170/558	(30,5)	
	Sí	38/54	(70,4)	16/54	(29,6)	
Rinovirus						0,002
	No	426/607	(70,2)	181/607	(29,8)	
	Sí	0/5	(0,0)	5/5	(100,0)	
*Haemophilus* spp.						0,112
	No	351/494	(71,1)	143/494	(28,9)	
	Sí	75/118	(63,6)	43/118	(36,4)	
*Moraxella* spp.						0,131
	No	412/587	(70,2)	175/587	(29,8)	
	Sí	14/25	(56,0)	11/25	(44,0)	
*Mycoplasma* spp.						<0,001
	No	407/569	(71,5)	162/569	(28,5)	
	Sí	19/43	(44,2)	24/43	(55,8)	
*Chlamydia* spp.						0,743
	No	419/601	(69,7)	182/601	(30,3)	
	Sí	7/11	(63,6)	4/11	(36,4)	
Adenovirus						>0,999
	No	424/610	(69,5)	186/610	(30,5)	
	Sí	2/2	(100,0)	0/2	(0,0)	
*Streptococcus pneumoniae*						0,221
	No	425/609	(69,8)	184/609	(30,2)	
	Sí	1/3	(33,3)	2/3	(66,7)	
Coronavirus						>0,999
	No	424/609	(69,6)	185/609	(30,4)	
	Sí	2/3	(66,7)	1/3	(33,3)	
Enterovirus						>0,999
	No	425/611	(69,6)	186/611	(30,4)	
	Sí	1/1	(100,0)	0/1	(0,0)	

Prueba X^2^ de independencia, prueba exacta de Fisher

En el modelo ajustado de la regresión de Poisson, se encontró que el tiraje subcostal se asoció independientemente con la hospitalización (1,4; IC_95%_: 1,05-1,88), mientras que, en la pospandemia, se asoció con hospitalización no prolongada (0,39; IC_95%_: 0,25 a 0,61) (cuadro 5).

**Cuadro 5.** Análisis multivariado de los factores asociados con la hospitalización prolongada en la población estudiada

Variable		Regresión sin ajustar			Regresión ajustada		
		RP	IC_95 %_	p	RP	IC_95 %_	p
Estacionalidad							
	Invierno	1,00			1,00		
	Otoño	1,25	0,91-1,72	0,162	1,14	0,83-1,57	0,418
	Primavera	1,21	0,85-1,70	0,289	1,23	0,87-1,75	0,247
	Verano	0,82	0,53-1,28	0,383	0,82	0,51-1,30	0,395
Periodo							
	Prepandemia	1,00			1,00		
	Pandemia	0,92	0,57-1,49	0,745	0,97	0,59-1,61	0,918
	Pospandemia	0,39	0,25-0,61	< 0,001	0,39	0,25-0,61	<0,001
Criterios OMS							
	No	1,00			1,00		
	Sí	1,32	0,99-1,75	0,057	1,13	0,72-1,77	0,600
Fiebre							
	No	1,00			1,00		
	Sí	1,26	0,98-1,62	0,070	1,22	0,82-1,79	0,324
Disnea							
	No	1,00			1,00		
	Sí	1,26	0,98-1,63	0,072	1,16	0,88-1,54	0,297
Vómitos							
	No	1,00			1,00		
	Sí	0,72	0,50-1,05	0,085	0,80	0,56-1,16	0,243
Odinofagia							
	No	1,00			1,00		
	Sí	0,44	0,15-1,27	0,128	0,52	0,18-1,47	0,219
Sibilancias							
	No	1,00			1,00		
	Sí	1,26	0,99-1,62	0,064	1,08	0,84-1,39	0,540
Tiraje subcostal							
	No	1,00			1,00		
	Sí	1,86	1,46-2,37	<0,001	1,40	1,05-1,88	0,022
Tiraje intercostal							
	No	1,00			1,00		
	Sí	1,65	1,14-2,40	0,009	1,26	0,85-1,85	0,244

RP: razón de prevalencia

### Estacionalidad y variación de las infecciones de las vías respiratorias inferiores durante, antes y después de la pandemia

En el periodo prepandemia, el 13,7 % (61/444) de las infecciones de las vías respiratorias inferiores fue de origen bacteriano, el 48,9 % (217/444), de etiología viral y, el 36,3 % (161/444), coinfecciones virus-bacteria. El otoño fue la estación en la que se presentó el mayor número de casos de infecciones de las vías respiratorias inferiores (36,93 %) (164/444).

Durante la pandemia, disminuyeron los casos de hospitalización por estas infecciones: se registraron 8 pacientes con infección bacteriana, 5 con infección viral y ningún caso de coinfección.

En la pospandemia, se reportaron 87,8 % (115/131) infecciones virales, 1,5 % (2/131) infecciones bacterianas y 9,9 % (13/131) coinfecciones. El otoño fue la estación en la que se presentaron más casos (36,64 %) (48/131) (cuadro 6).

**Cuadro 6:** Estacionalidad de los agentes patógenos respiratorios detectados en la población estudiada

Periodo			Agente patógeno		p*
			n (%)		
Prepandemia		Bacterias (n = 39)	Virus (n = 139)	Coinfección (n = 106)	
	Invierno	14/72 (19,4)	29/72 (40,3)	29/72 (40,3)	< 0,001
	Otoño	4/105 (3,8)	55/105 (52,4)	46/105 (43,8)	
	Primavera	11/63(17,5)	27/63 (42,9)	25/63 (39,7)	
	Verano	10/44 (22,7)	28/44 (63,6)	6/44 (13,6)	
Pandemia		Bacterias (n = 8)	Virus (n = 5)	Coinfección (n = 0)	
	Invierno	1/1	0	0	0,359
	Otoño	1/4	2/4	0	
	Primavera	5/6	1/6	0	
	Verano	1/3	2/3	0	
Pospandemia		Bacterias (n = 2)	Virus (n = 104)	Coinfección (n = 12)	
	Invierno	0/31	30/31 (96,8)	1/31	< 0,001
	Otoño	1/44	43/44 (97,7)	0/44	
	Primavera	1/38	28/38 (73,7)	9/38	
	Verano	0/5	3/5 (60,0)	2/5	

Prueba de X^2^ de independencia y prueba exacta de Fisher

## Discusión

EI objetivo de este estudio fue determinar la frecuencia y los factores asociados con los microorganismos en niños hospitalizados por infección de las vías respiratorias inferiores, encontrándose que la etiología viral fue más frecuente (59,7 %) (365/612) en comparación con la bacteriana (11,8 %) (72/612). El agente etiológico más frecuente fue el virus sincitial respiratorio (33,7 %) (206/612). Resultó llamativo que la suma de frecuencias de virus de la influenza Ay de la influenza B fue del 35,1 % (215/612).

En las etapas de prepandemia y pospandemia, dichas infecciones respiratorias fueron frecuentes en el otoño y la etiología más frecuente fue la viral, con predominancia del virus sincitial respiratorio en la prepandemia y del virus de la influenza A en la pospandemia. Además, se observó con frecuencia la coinfección del virus sincitial respiratorio y otros microorganismos, tanto virales como bacterianos.

En el periodo anterior a la pandemia, se observó que la prevalencia de las infecciones virales (48,9 %) (217/444) estaba asociada con un elevado índice de coinfecciones (36,3 %) (161/444). Por ejemplo, en un estudio descriptivo de la República de Cabo Verde, antes de la pandemia, se encontró que los virus patógenos más frecuentes fueron rinovirus o enterovirus, el de la influenza A y el virus sincitial respiratorio. Agregado a lo anterior, las coinfecciones de múltiples agentes patógenos se detectaron en el 20,6 % de los casos mediante la PCR en tiempo real ^13^. Al comparar este resultado con el periodo de la pandemia, se registró una disminución mayor del 90 % de los casos de infección de las vías respiratorias inferiores. Esta disminución también se observó en otros países como Francia, Finlandia, Estados Unidos, Brasil, Marruecos y China ^13^^-^^16^.

En un estudio en Australia entre el 2015 y el 2020, se evidenció que, después de las medidas de prevención de la COVID-19, más específicamente entre abril y junio del 2020, los casos por el virus sincitial respiratorio habían sido el 94,3 % menos de lo esperado en comparación con la tendencia de los años anteriores (2015-2019). En consecuencia, disminuyeron las admisiones a las unidades de cuidado intensivo, ya que este virus respiratorio es el que más se asocia con la hospitalización por bronquiolitis en la población pediátrica. En el mencionado estudio, también atribuyen esta reducción a las medidas de distanciamiento social y al lavado de manos que daña la envoltura lipídica que recubre el virus ^17^.

Perú fue uno de los países de la Latinoamérica que presentó más rápida respuesta y medidas más radicales frente a la pandemia por la COVID-19, como el aislamiento social obligatorio y prolongado, el uso de mascarilla y la promoción del lavado de manos ^12^.

En Perú, en un estudio retrospectivo llevado a cabo durante la pandemia en 342 pacientes con infección respiratoria aguda y con prueba de COVID-19 negativa mediante una prueba de PCR en tiempo real, se reportó: rinovirus en el 54,4 % (93/171) de los casos, influenza en el 23,9 % (41/171) y virus sincitial respiratorio en el 14 % (24/171). Estos datos son similares a los reportados en otros países ^18^. Se requieren evaluaciones con muestras más grandes de población con el fin de caracterizar mejor la dinámica de las frecuencias de las etiologías virales de las infecciones respiratorias agudas durante la pandemia en el Perú.

El periodo pospandemia se caracteriza por el fin del aislamiento social y, en el contexto de los niños, por la reapertura de los colegios y el fin de la obligatoriedad del uso de mascarillas. En el presente estudio se observa una baja frecuencia de infecciones bacterianas (1,52 %) (2/131) y de coinfección (7,6 %) (13/131), mientras que la frecuencia de los cuadros virales empieza a regresar a sus valores prepandémicos, con un 87,8 % (115/131) de infecciones de las vías respiratorias inferiores. Este resultado también se puede observar en otros países, como Austria, Nueva Zelanda, Reino Unido, Japón y China, en donde, después de la flexibilización de las medidas de protección contra la COVID-19, se demostró un aumento de la incidencia del rinovirus, a diferencia del presente estudio, en la cual se encontró predominantemente una mayor frecuencia de la influenza A. Una de las hipótesis para el incremento de rinovirus es que sus características estructurales, como la de no tener envoltura, lo hacen menos susceptible a la inactivación por el lavado de las manos con agua y jabón ^17^^,^^19^^-^^21^. Dado que la información local no es suficiente, se requieren evaluaciones de poblaciones más numerosas para determinar la frecuencia de los virus patógenos posteriores a la pandemia.

El otoño fue la estación del año con mayor cantidad de hospitalizaciones por infecciones de las vías respiratorias inferiores (p <0,001) y el virus sincitial respiratorio fue el agente patógeno más frecuente en este estudio (n = 206/612). Estos resultados concuerdan con los de otros reportes ^17^. Todavía no hay una respuesta unánime que explique cómo la estacionalidad aumenta la frecuencia de estos casos, pero factores como las bajas temperaturas y la disminución de la humedad se han mencionado como predisponentes a infecciones respiratorias ^22^.

La presencia de tiraje subcostal e intercostal tuvo una asociación significativa con la prolongación del tiempo de hospitalización (p < 0,001 y p = 0,020, respectivamente). Según McCollum *et al*., existe una asociación entre tirajes y neumonía en los niños mayores de dos años, lo que explicaría la mayor gravedad de los casos, la cual se traduce en mayor tiempo de hospitalización ^23^.

Otro factor que se asoció con la estancia hospitalaria prolongada fue la detección de *M. pneumoniae*. Resulta interesante que este hallazgo ha sido reportado en pacientes con neumonía lobar con tapones mucosos inducidos por *M. pneumoniae*^24^. También, se ha reportado que la coinfección entre virus patógenos y *M. pneumoniae* está asociada con prolongación del tiempo de hospitalización; llama la atención que los reportes relacionan esta asociación con la coinfección por adenovirus; esto se ha justificado por la respuesta inmunitaria intensificada, mediante la cual la liberación de citocinas induce daño de las células epiteliales intestinales ^17^^,^^25^.

Hasta donde se tiene registro, este es el primer estudio peruano en el que se describen los agentes etiológicos más frecuentes en los casos de coinfección en la población pediátrica hospitalizada por infección de las vías respiratorias inferiores. Se reportó una tasa del 19,28 % (118/612) de cuadro clínico de coinfección, que es un porcentaje que concuerda con el de estudios internacionales, los cuales presentan tasas que varían del 14,1 al 22,1 %. Además, el virus sincitial respiratorio fue el que más se asoció con los cuadros de coinfección (figura 3), hallazgo similar a lo informado en otros estudios ^17^^,^^26^.

En la literatura se reporta gravedad de la coinfección del virus sincitial respiratorio con bacterias patógenas como *H. influenzae*, *S. penumoniae* o ambas. En la coinfeccion de *S. pneumoniae* y el virus sincitial respiratorio, se ha encontrado interacción positiva entre ambos microorganismos, la cual produce potenciación del virus y predisposición a desarrollar enfermedad neumocócica invasiva. Además, los perfiles del microbioma dominados por *S. pneumoniae* o *H. influenzae* en niños, se han asociado con respuestas proinflamatorias mucosas potenciadas ^27^. Este resultado es llamativo, dado que esta detección se ha asociado con hospitalización prolongada por la mayor gravedad de los casos. Aunque en el presente estudio no se evidenció esta asociación, se necesitan estudios prospectivos en Perú que incluyan mayor población para conocer mejor las características de las coinfecciones en los pacientes pediátricos con infección de las vías respiratorias inferiores.

En el estudio publicado por Mandelia *et al*., se evidenció que la coinfección en la población pediátrica era más frecuente que en la población adulta, especialmente en los niños menores de 5 años. En el 2020, se encontró que la población pediátrica presentaba una tasa de coinfección del 35 % (2068/5906) en comparación con el 5,8 % (270/4591) en los adultos. Según Mandelia *et al*., esta diferencia podría deberse a la diversidad de agentes patógenos exclusivos de estos grupos de edad y a la diferencia en la forma de exposición a los virus ^21^.

No se conocen con certeza las consecuencias de la coinfección, aunque en un estudio japonés, se encontró evidencia de que las coinfecciones del virus sincitial respiratorio con otros virus, como el rinovirus, el virus de la parainfluenza o el metapneumovirus, se asociaban con un mayor riesgo de neumonía, en comparación con la infección por cualquiera de estos virus individualmente ^28^. Esto podría justificar el aumento del tiempo de hospitalización en nuestro estudio.

En cuanto a las limitaciones, en el presente estudio, se utilizaron dos pruebas de ensayo múltiple de reacción en cadena de la polimerasa para detectar los virus y las bacterias más frecuentemente asociadas con infecciones respiratorias; sin embargo, existe la posibilidad de que haya otros microorganismos que no fueron detectados con ninguna de las dos pruebas.

En los distritos y provincias del departamento de Lima, durante el periodo de pandemia, las medidas de aislamiento social fueron estrictas y de carácter obligatorio, lo que pudo resultar en una disminución de atenciones en urgencias y, consecuentemente, en un menor número de admisiones en el servicio de hospitalización. Los hospitales privados reciben a los pacientes de forma pasiva, así que los hallazgos de este estudio no se extrapolan a la población de Lima porque el muestreo fue no sistemático.

Como conclusión, en este estudio se encontró que los agentes etiológicos virales fueron predominantes en comparación con las bacterias, antes y después de la pandemia, siendo más prevalentes en el periodo pospandemia. El virus sincitial respiratorio fue el agente etiológico más frecuente y se asoció con coinfecciones y estancia hospitalaria prolongada, dos características que juntas aumentan la morbilidad y gravedad de los casos de infección de las vías respiratorias inferiores en los pacientes pediátricos.

Es importante mencionar la asociación entre la presencia de tirajes y el mayor tiempo de hospitalización, lo que demuestra la importancia de la identificación temprana de este signo, además de un rápido manejo para evitar futuras complicaciones. Son necesarios más estudios, con poblaciones más numerosas, para demostrar la coherencia de los hallazgos en los años posteriores a la pandemia.
